# Augmented Innate and Adaptive Immune Responses Under Conditions of Diabetes–Filariasis Comorbidity

**DOI:** 10.3389/fimmu.2021.716515

**Published:** 2021-09-10

**Authors:** Joy Manohar Sibi, Viswanathan Mohan, Saravanan Munisankar, Subash Babu, Vivekanandhan Aravindhan

**Affiliations:** ^1^Department of Genetics, Dr A. L. Mudaliar Post Graduate Institute of Basic Medical Sciences (ALM PG IBMS), University of Madras, Chennai, India; ^2^Madras Diabetes Research Foundation and Dr. Mohan’s Diabetes Specialties Centre, Chennai, India; ^3^National Institute of Health–International Centre for Excellence in Research, National Institute for Research in Tuberculosis, Chennai, India

**Keywords:** diabetes, filariasis, TLR, immunomodulation, Th cell, inflammation

## Abstract

Metainflammation, as seen in chronic diabetes subjects, impairs immunity and increases the susceptibility to infections. In the present study, the effect of diabetes on immune response against filariasis was studied. Both toll-like receptor (TLR)-mediated and crude antigen-induced immune responses were quantified, in whole blood cultures from filariasis-infected subjects (LF+), with and without diabetes. Blood cultures were stimulated with TLR ligands (TLR2 and TLR4) or filarial antigen or were left unstimulated (control) for 18 h. Cytokine, chemokine, and defensin secretion was quantified by ELISA. Expression of HLA-DR, B7-1, B7-2, activation marker (CD69), and Th (Th1, Th2, Th17, and Th9) phenotypes was quantified by flow cytometry. Expression of immunomodulatory effectors (Cox-2, HO-1, IDO-1, and p47Phox) and Th-polarizing transcription factors (T-bet, GATA3, and ROR-γt) was quantified by quantitative PCR. Secretion of IL-27, IL-1Ra, IL-12, IL-33, IL-9, and SDF-1 was increased under diabetes conditions with increased Th9 polarization and increased expression of Cox-2 and IDO. Overall, diabetes was found to augment both TLR-mediated and antigen-induced inflammation, which can promote chronic pathology in LF+ subjects.

## Introduction

Filarial infections, unlike viral and bacterial infections, induce immunomodulation, rather than inflammation (1). Murine studies have shown significant protection against both forms of diabetes (type 1 and type 2) by filarial pre-infections (2–4). Previously, we have shown a decreased prevalence of filariasis among both T1DM (5) and T2DM (6) subjects in the South Indian population. Serum cytokine profiling in these subjects showed significant downregulation of pro-inflammatory cytokines [interleukin-6 (IL-6), tumor necrosis factor-alpha (TNF-α), and granulocyte–macrophage-colony stimulating factor (GM-CSF)] and upregulation of anti-inflammatory cytokine [tumor growth factor-beta (TGF-β)], in filarial-positive diabetic subjects (6). Interestingly, this effect was specific only to T1DM and T2DM and was not seen in those with coronary artery disease (CAD) (7). These observations were later replicated in other Asian countries like China (8) and Indonesia (9).

The relationship between filariasis and diabetes is bi-directional (10). While childhood filarial infection can dampen inflammation and can confer protection against diabetes, the effect of diabetes on filariasis-induced immune response is largely unknown. This is relevant in endemic zones wherein at least some of the subjects with lymphatic filariasis (LF) would develop diabetes, during the course of infection. T2DM in general, weakens the immune system and makes the patients more susceptible to infections (11). Immune response against filariasis, in general, can be broadly classified into innate and adaptive immune responses. Toll-like receptors (TLRs) serve as the first line of defense mechanism, bridging the innate and adaptive arms of the immune responses (12). They are abundantly present in cells of the innate immune system, which include macrophages, dendritic cells, neutrophils, and eosinophils (13). Previously, several filarial antigens were shown to bind directly to TLRs and activate them (14, 15). Upon TLR ligation, the professional antigen-presenting cells (APCs) that include macrophages, dendritic cells, and B cells undergo activation followed by the secretion of cytokines and chemokines (16). The cytokines secreted by the innate immune cells can be broadly classified into (1) type 1 interferons (interferon-α and β), (2) pro-inflammatory cytokines (TNF-α, IL-1β, IL-6, and GM-CSF), and (3) anti-inflammatory cytokines (IL-10, TGF-β, IL-1Ra, IL-35, and IL-27) (17). TLR ligation also upregulates the antigen processing and presentation machinery, which includes MHC (HLA) and co-stimulatory (B7-1/CD80 and B7-2/CD86) molecules (18). Furthermore, certain cell-type-specific effector functions like secretion of antibodies by B cells (19), defensin by neutrophils (20), and matrix-metalloproteinases (MMPs) by macrophages (21) are also augmented. TLR-mediated expression of Cyclooxygenase-2 (Cox-2), Heme Oxygenase-1 (HO-1), and Indoleamine-2,3-Dioxygenase (IDO) constitute a immunoregulatory circuit, which controls filariasis-mediated immunomodulation (22). The net result is the recruitment and activation of T cells, which marks the transition from innate to adaptive immune response (12).

In contrast to the cells of innate immune system, that detect pathogens through TLRs, T cells are the workhorses of the adaptive immune system, largely depending on surface T-cell receptors (TCRs), for detecting the pathogens (23). While the major function of CD4^+^ helper T (Th) cells is to regulate other cells, CD8^+^ cytotoxic T (Tc) cells are mainly involved in the elimination of virally infected cells and tumor cells (24). In contrast to the innate immune cytokines, the adaptive immune cytokines are secreted by T cells (effector cytokines) and APCs (polarizing cytokines) (25). Upon engagement of the TCRs and antigen co-receptor (CD28), the Th cells undergo differentiation into various sub-types depending on the polarizing cytokines secreted by the APCs (25). Depending on cytokine secretion, Th cells are classified into Th1 (IL-12, IFN-γ, and IL-2), Th2 (IL-33, IL-4, IL-5, and IL-13), Th9 (IL-9), and Th17 (IL-23, IL-17, and IL-17F) sub-types (26). T cell-mediated immune responses during filarial infection largely depend on the phase of the infection: (1) acute phase—skewed towards the Th2 response; (2) chronic phase—skewed towards “modified Th2 response”, with Tregs playing a more prominent role compared to Th2 cells; (3) chronic pathology phase—a drastic shift from “modified Th2” response to pro-inflammatory “Th1/Th17” response takes place and happens only in those who develop lymphatic pathology (22). Thus, in the present study, we looked at both TLR-mediated innate and filarial antigen-induced adaptive immune responses in LF+ subjects, both with and without diabetes. Cytokine/chemokine secretion, expression of immunomodulatory enzymes, upregulation of MHC and co-stimulatory molecules, T-cell activation, Th polarization, and expression of T-cell polarizing transcription factors were quantified.

## Materials and Methods

### Study Subjects

This study is a follow-up of our previous publication wherein we reported decreased prevalence of LF among diabetic subjects compared to control subjects (6). As a continuation of this study, 1,001 outpatients visiting Dr. Mohan’s Diabetes Specialties Centre, Chennai, India, were screened for LF. Out of 1,001, only 12 were found to be positive for LF antigen (1.2%). None of them had any clinical symptoms of LF (DM-LF+). As controls, eight normal glucose-tolerant (non-diabetic) LF+ subjects were included (NGT-LF+). The control subjects were recruited from the healthy volunteers who accompanied the diabetes patients and underwent OGTT testing, as part of this study. Institutional ethical committee approval from the Madras Diabetes Research Foundation Ethics Committee was obtained (Ref No-MDRF-EC/SOC/2009//05) and written informed consent was obtained from all the study subjects. The study was conducted as per the Declaration of Helsinki, following STROBE guidelines.

### Inclusion and Exclusion Criteria

Only subjects who were LF+ (as determined by TropBio) were included in the study. None of the subjects showed any symptoms of lymphatic filariasis at the time of recruitment. The exclusion criteria were patients with type 1 diabetes and those with a previous diagnosis of urolithiasis, liver cirrhosis, congestive heart failure, chronic lung diseases, chronic infections, or viral hepatitis.

### Diagnosis of T2DM

The diagnosis was done following WHO guidelines. Subjects were grouped as Control (NGT) based on the Oral Glucose Tolerance Test (OGTT) and as diabetes based on previous history (https://www.who.int/diabetes/publications/Definition%20and%20diagnosis%20of%20diabetes_new.pdf) (ISBN 978-92-4-159493-6).

### Detection of Bancroftian LF

To quantify the filarial antigen levels, sera were analyzed using the *W. bancrofti* Og4C3 antigen-capture ELISA (Tropbio, Australia) according to the manufacturer’s instructions.

### Preparation of Filarial Antigen

Crude antigen extract from *Brugia malayi* antigen (BmA) was prepared by extracting somatic antigens from the live L3 larvae in 1× PBS, as described previously (27). The extract was concentrated, quantified, and stored at −80°C. The endotoxin levels as determined by the limulus amoebocyte lysate assay (QCL-1000 kit; BioWhittaker) was below the detection limit in these preparations. It is important to note that both *W. bancrofti* and *Brugia malayi* share several overlapping antigens, and immune cells from patients infected with *W. bancrofti* mount a strong immune response against *Brugia malayi* antigens during rechallenge experiments (28).

### Peripheral Blood Leukocyte Cultures

Whole blood cultures from the study subjects were done as described previously (29). Whole blood was collected in EDTA-coated tubes. After centrifugation, the packed cell volume was diluted with RPMI medium (1:1 ratio) containing 10% FCS and was used for *in vitro* culture. Cells were stimulated with TLR2 ligand-PAM3CSK4 (100 ng/ml) (Invivogen, USA) or TLR4 ligand-LPS (100 ng/ml) (Invivogen, USA) or were left unstimulated for 24 h in parallel cultures. For antigen stimulation, cultures were stimulated with crude extract of BmA (10 µg/ml) for 24 h. The supernatants were harvested for cytokine/chemokine estimation. The cell pellets were solubilized in RNAzol and were stored at −80°C. Cell pellets from parallel cultures were treated with golgi stop for 6 h, washed, and stained for flow cytometry.

### Measurement of Cytokines and Chemokines by ELISA

The levels of cytokines (TNF-α, IL-6, IL-1β, GM-CSF, IL-10, TGF-β, IL-27, IL-1Ra, IFN β, IL-12p70, IFN-γ, IL-2, IL-23, IL-33, IL-4, IL-17, and IL-9), chemokines (SDF-1, IL-8, MCP-1, RANTES, and IP-10) and α-defensin-1 in the cell supernatant were quantified using ELISA (R&D, USA) following the manufacturer’s protocol. IL-35 was estimated using pre-coated ELISA plates (Immunoconcept). The lower detection limits were as follows: TNF-α = 1.95 pg/ml, IL-6 = 0.59 pg/ml, IL-1β = 0.24 pg/ml, GM-CSF = 0.67 pg/ml, IL-10 = 9.76 pg/ml, TGF-β = 1.95 pg/ml, IL-35 = 6.25 pg/ml, IL-27 = 19.53 pg/ml, IL-1Ra = 0.144 pg/ml, IL-12 = 1.95 pg/ml, IFN-γ = 1.17 pg/ml, IL-33 = 0.016 pg/ml, IL-4 = 1.9 pg/ml, IL-17, IL-9, SDF-1 = 107.2 pg/ml, IL-8 = 1.98 pg/ml, IP-10 = 0.97 pg/ml, and α-defensin = 62.50 pg/ml. The CV was found to be <10%. The list of various monoclonal antibodies used in ELISA is provided in **Table S1**.

### Flow Cytometry

Cells were stained with fluorochrome-conjugated monoclonal antibodies specific for CD3, CD4, CD19, CD14, CD69, HLA-DR, CD80, and CD86. They were then permeabilized with saponin (0.01%), stained with antibodies specific for IFN γ, IL-4, IL-17A, and IL-9, and were analyzed on FACS Canto (BD Biosciences, USA). The lymphocytes, monocytes, and granulocytes were first gated based on the FSC *vs*. SSC plot. Monocytes and B cells were further gated based on CD14 and CD19 expression, respectively. T-helper cells were gated based on CD3 and CD4 expression. The expression of cytokines and other effector molecules within the gated population was analyzed as illustrated in **Figures S1** and **S2**. Both the percentage and mean fluorescence intensity (MFI) of the gated cell population were quantified using FlowJo software, version v10 (BD Biosciences, USA). The list of various monoclonal antibodies used in flowcytometry is provided in **Table S2**.

### Real-Time PCR Analysis

RNA extraction from the stored samples was carried out using RNeasy Mini Kit (Qiagen). The quality and quantity of the extracted RNA was quantified using nanodrop. One microgram of RNA was converted to cDNA using reverse transcription and real-time PCR was performed using TaqMan probes (Applied Biosystems, USA) specific for Cox-2, IDO, Phox P47, HO-1, T-bet, GATA-3, and ROR-γt. 18S rRNA was used as a house-keeping control. Gene expression levels (normalized to 18S rRNA) were analyzed using the StepOnePlus RT-PCR system (Applied Biosystems, Foster City, CA, USA) and 2^–ΔΔCt^ was calculated for all samples with unstimulated sample values as reference. All the probes used in these studies were purchased from Applied Biosystems, and the list of various probes used is provided in **Table S3**.

### Statistical Analysis

Mann–Whitney *U* test was used for comparing NGT *versus* DM groups. Multiple comparisons were corrected using Holm’s correction. All the analyses were done using GraphPad Prism version 5.0 (GraphPad Software, USA). *p*-value less than 0.05 was considered significant.

## Results

### Clinical Characteristics of the Study Subjects

**Table S4** shows the clinical characteristics of the study groups. As can be seen in the table, DM-LF+ subjects had significantly increased BMI, glycemic parameters (FPG, PPPG, and HbA1c), total cholesterol, and total triglyceride lipids, compared to NGT-LF+ subjects.

### Effect of Diabetes on the TLR-Induced Secretion of Pro- and Anti-Inflammatory Cytokines, Type 1 Interferons, and Defensins in LF+ Subjects

**Figure 1:**
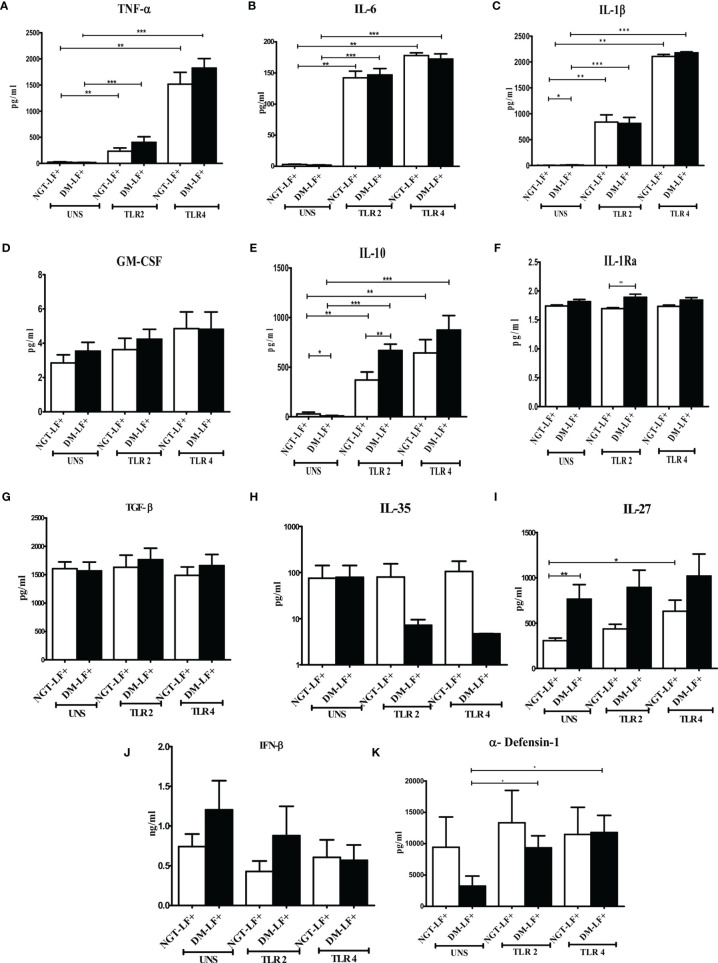
Effect of diabetes on the TLR-induced secretion of pro- and anti-inflammatory cytokine type 1 interferons and defensins in LF+ subjects. Bar graph showing Unstimulated (UNS), TLR2-, and TLR4-induced secretion of TNF-α **(A)**, IL-6 **(B)**, IL-1β **(C)**, GM-CSF **(D)**, IL-10 **(E)**, IL-1Ra **(F)**, TGF-β **(G)**, IL-35 **(H)**, IL-27 **(I)**, IFN-β **(J)**, and α-defensin **(K)** in the supernatants of blood cultures in NGT-LF+ and DM-LF+ subjects. Statistical significance was determined by non-parametric Mann–Whitney *U* test and *p* < 0.05 was considered significant. *p < 0.05; **p < 0.01; ***p < 0.001.

**Figure 1** shows the TLR-induced secretion of pro- (TNF-α, IL-6, IL-1β, and GM-CSF) and anti-(IL-10, TGF-β, IL-1Ra, IL-35, and IL-27) inflammatory cytokines, type 1 interferons (IFN-β), and defensins (α-defensin-1) in the study groups. At the basal level, IL-1β and IL-27 levels were significantly higher in the DM-LF+ group compared to the NGT-LF+ group. TLR2 stimulation resulted in the secretion of TNF-α, IL-6, IL-1β, and IL-10. TLR4 stimulation resulted in the secretion of TNF-α, IL-6, IL-1β, IL-10, and IL-27. Significant difference was seen in the secretion of IL-10 and IL-1Ra between the NGT-LF+ and DM-LF+ groups. TLR2 and TLR4 induced α-defensin-1 secretion only in the DM-LF+ group.

### Effect of Diabetes on the TLR-Induced Secretion of Adaptive Immune Cytokines and Chemokines in LF+ Subjects

**Figure 2:**
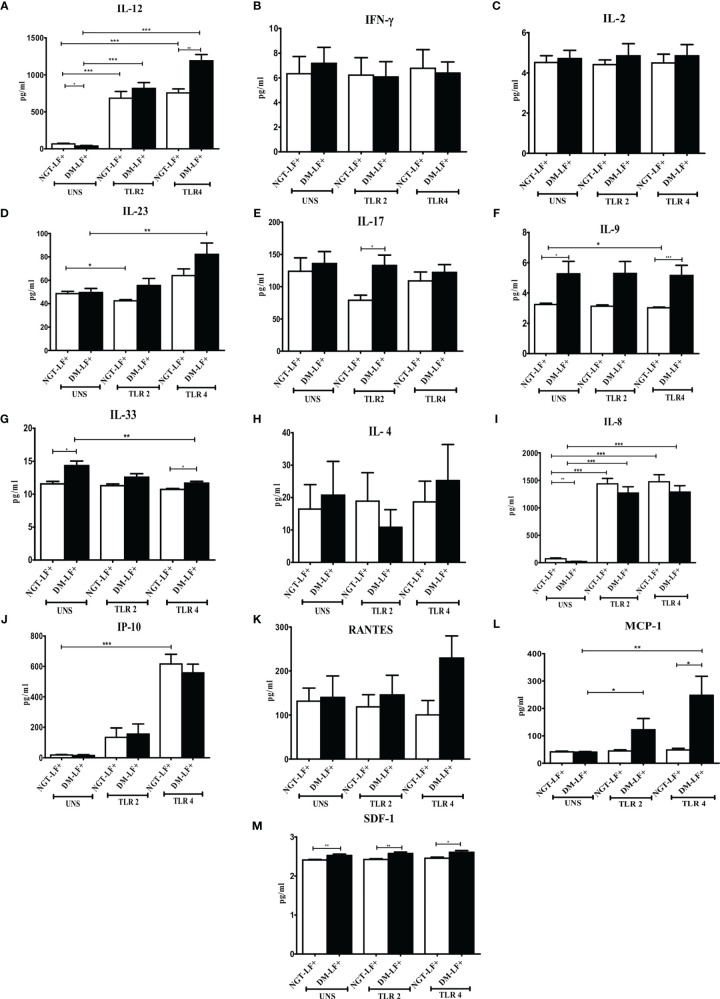
Effect of diabetes on the TLR-induced secretion of adaptive immune cytokines and chemokines in LF+ subjects. Bar graph showing Unstimulated (UNS), TLR2-, and TLR4-induced secretion of IL-12 **(A)**, IFN-γ **(B)**, IL-2 **(C)**, IL-23 **(D)**, IL-17 **(E)**, IL-9 **(F)**, IL-33 **(G)**, IL-4 **(H)**, IL-8 **(I),** IP-10 **(J)**, RANTES **(K)**, MCP-1 **(L)**, and SDF-1 **(M)** in the supernatants of blood cultures in NGT-LF+ and DM-LF+ subjects. Statistical significance was determined by non-parametric Mann–Whitney *U* test and *p* < 0.05 was considered significant. *p < 0.05; **p < 0.01; ***p < 0.001.

**Figure 2** shows the TLR-induced secretion of adaptive immune cytokines (IL-12, IFN-γ, IL-2, IL-23, IL-17, IL-9, IL-33, and IL-4) and chemokines (IL-8, IP-10, RANTES, MCP-1, and SDF-1) in the study groups. TLR2 stimulation resulted in the secretion of IL-12 and IL-8 and decreased the secretion of IL-23. TLR4 stimulation resulted in the secretion of IL-12, IL-23, IL-8, and IP-10. The basal secretion of IL-12 and IL-8 was decreased while that of IL-17, IL-9, IL-33, and SDF-1 was augmented in the DM-LF+ compared to the NGT-LF+ group. TLR2 and TLR4 induced MCP-1 and downregulated IL-33 secretion only in the DM-LF+ group.

### Effect of Diabetes on the TLR-Induced Expression of MHC and Costimulatory Molecules in LF+ Subjects

**Figure S3** shows the expression of HLA-DR, CD80, and CD86 in B cells, monocytes, and granulocytes in the study groups. No significant difference was seen in the expression of these molecules between the groups.

### Effect of Diabetes on the TLR-Induced Expression of Immunomodulatory Effectors in LF+ Subjects

**Figure 3:**
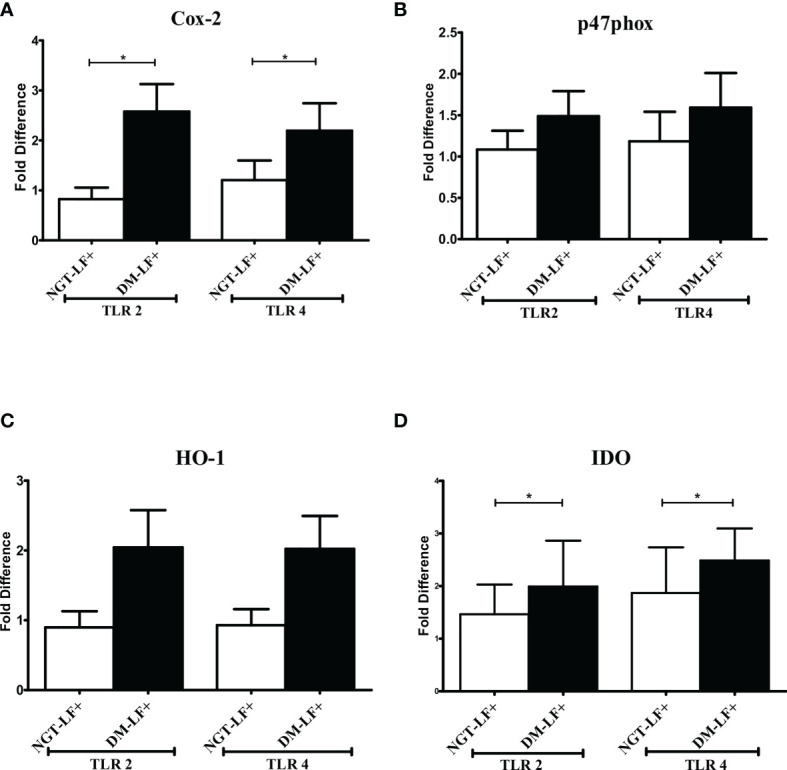
Effect of diabetes on the TLR-induced expression of immunomodulatory effectors in LF+ subjects. Bar graph showing TLR2- and TLR4-induced expression of Cyclooxygenase (Cox)-2 **(A)**, p47Phox **(B)**, Heme Oxygenase (HO)-1 **(C)**, and Indoleamine 2,3-dioxygenase (IDO) **(D)** in the immune cells in NGT-LF+ and DM-LF+ subjects. Statistical significance was determined by non-parametric Mann–Whitney *U* test and *p* < 0.05 was considered significant. *p < 0.05. NGT, normal glucose tolerance; DM, diabetes mellitus.

**Figure 3** shows the TLR-induced expression of immunomodulatory effectors (Cox-2, HO-1, Phox47, and IDO), in the study groups. TLR-induced expression of Cox-2 and IDO was significantly augmented in the diabetic group (DM-LF+).

### Effect of Diabetes on the Antigen-Induced Secretion of Pro- and Anti-Inflammatory Cytokines, Type 1 Interferons, and Defensins in LF+ Subjects

**Figure 4:**
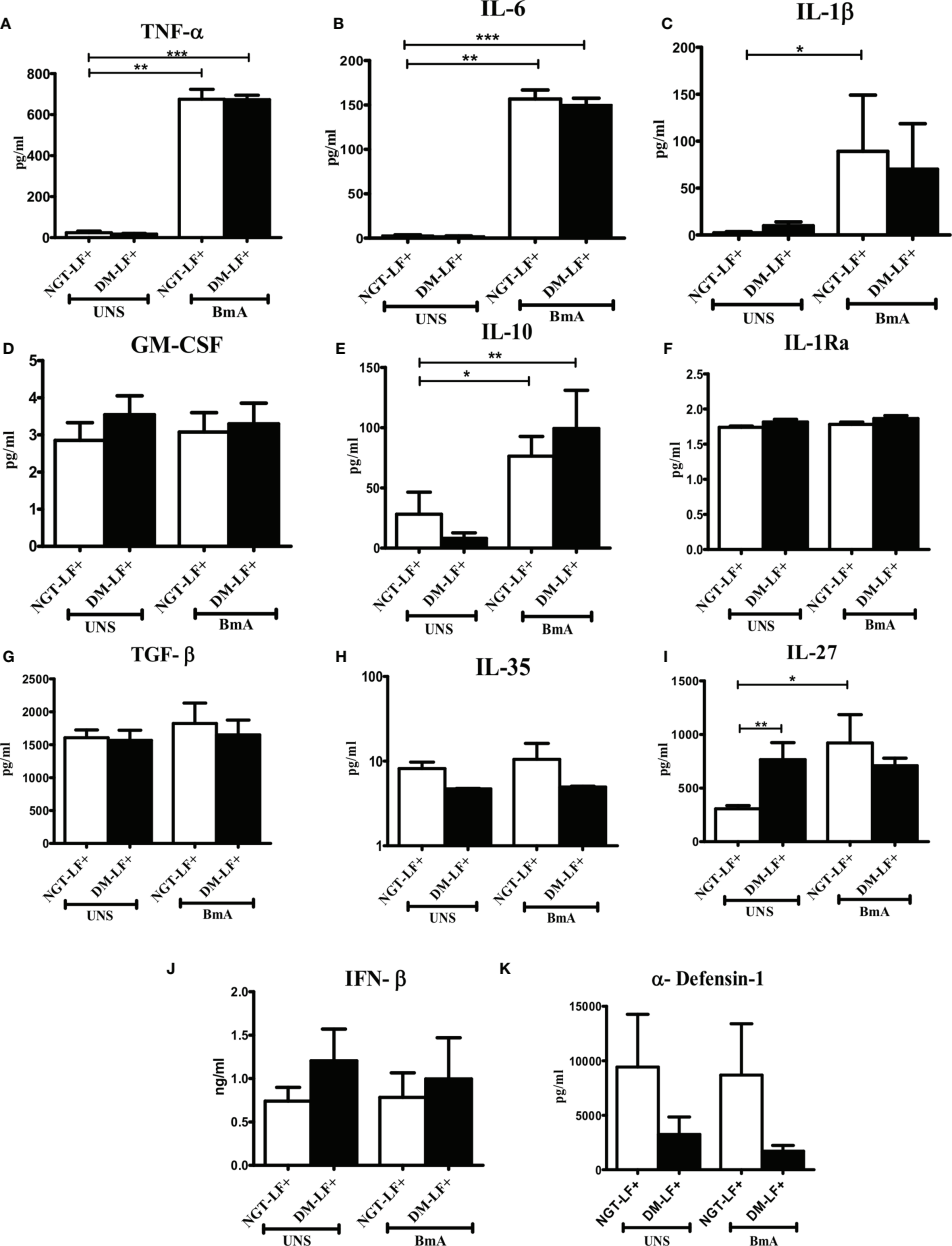
Effect of diabetes on the antigen-induced secretion of pro- and anti-inflammatory cytokines, type 1 interferons, and defensins in LF+ subjects. Bar graph showing Unstimulated (UNS) and filarial antigen (BmA)-induced secretion of TNF-α **(A)**, IL-6 **(B)**, IL-1β **(C)**, GM-CSF **(D)**, IL-10 **(E)**, IL-1Ra **(F)**, TGF-β **(G)**, IL-35 **(H)**, IL-27 **(I)**, IFN-β **(J)**, and α-defensin **(K)** in the supernatants of blood cultures in NGT-LF+ and DM-LF+ subjects. Statistical significance was determined by non-parametric Mann–Whitney *U* test and *p* < 0.05 was considered significant. *p < 0.05; **p < 0.01; ***p < 0.001.

**Figure 4** shows the antigen-induced secretion of pro- (TNF-α, IL-6, IL-1β, and GM-CSF) and anti- (IL-10, TGF-β, IL-1Ra, IL-35, and IL-27) inflammatory cytokines, and type 1 interferon (IFN-β) and defensin (α-defensin-1) cytokines in the study groups. BmA stimulation resulted in the secretion of TNF-α, IL-6, IL-1β, IL-10, and IL-27. No significant difference was seen in the secretion of these cytokines between the groups. Thus, with respect to pro- and anti-inflammatory cytokines, antigen-induced secretion of these cytokines remains largely unaffected by diabetes.

### Effect of Diabetes on the Antigen-Induced Secretion of Adaptive Immune Cytokines and Chemokines in LF+ Subjects

**Figure 5:**
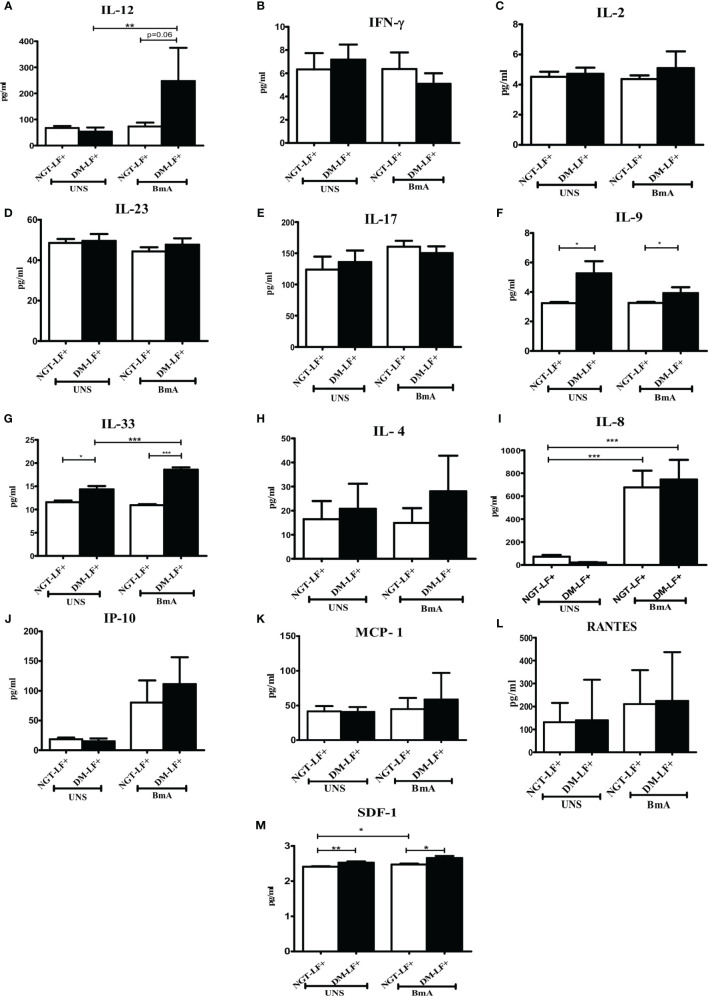
Effect of diabetes on the antigen-induced secretion of adaptive immune cytokines, and chemokines in LF+ subjects. Bar graph showing Unstimulated (UNS) and filarial antigen (BmA)-induced secretion of IL-12 **(A)**, IFN-γ **(B)**, IL-2 **(C)**, IL-23 **(D)**, IL-17 **(E)**, IL-9 **(F)**, IL-33 **(G)**, IL-4 **(H)**, IL-8 **(I),** IP-10 **(J)**, RANTES **(K)**, MCP-1 **(L)**, and SDF-1 **(M)** in the supernatants of blood cultures in NGT-LF+ and DM-LF+ subjects. Statistical significance was determined by non-parametric Mann–Whitney *U* test and *p* < 0.05 was considered significant. *p < 0.05; **p < 0.01; ***p < 0.001.

**Figure 5** shows the antigen-induced secretion of adaptive immune cytokines (IL-12, IFN-γ, IL-2, IL-23, IL-17, IL-9, IL-33, and IL-4) and chemokines (IL-8, IP-10, RANTES, MCP-1, and SDF-1) in the study groups. BmA stimulation resulted in the secretion of IL-8. BmA-induced secretion of IL-12 and IL-33 was seen only in the DM-LF+ group.

### Effect of Diabetes on T-Cell Activation, Th Polarization, Expression of Th Master Regulators, and Immunomodulatory Enzymes in LF+ Subjects

**Figure 6:**
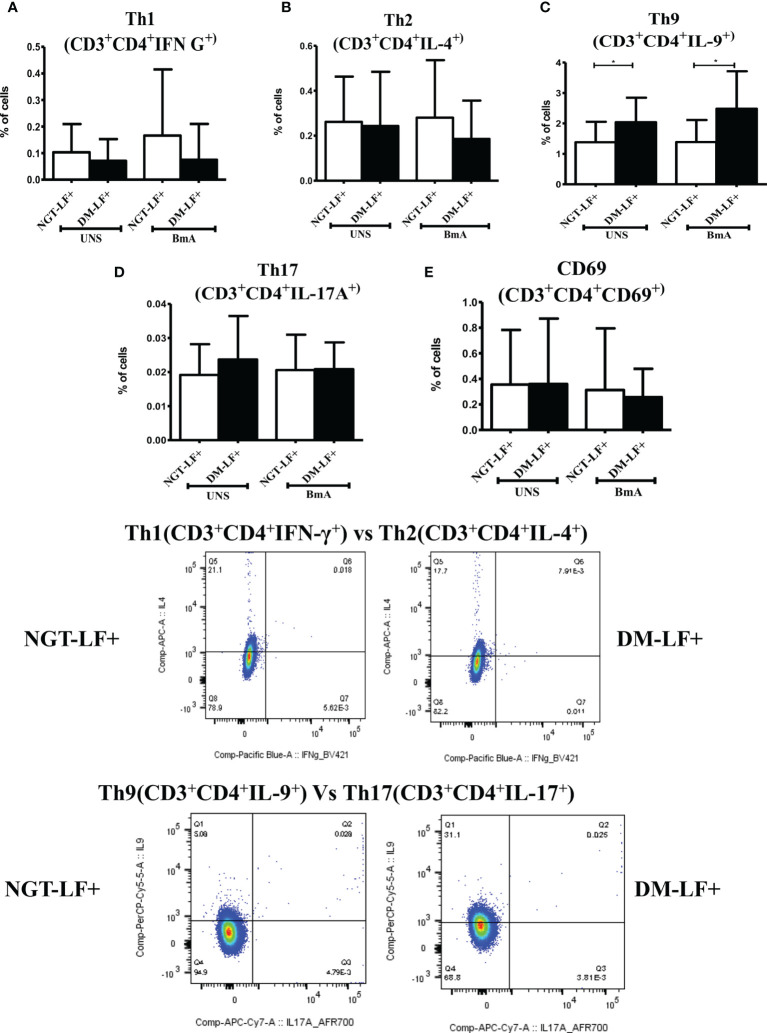
Effect of diabetes on T-cell activation and Th polarization in LF+ subjects. Bar graph showing Unstimulated (UNS) and filarial antigen (BmA)-induced Th1 **(A)**, Th2 **(B)**, Th9 **(C)**, Th17 **(D)**, and activated T cells **(E)** in NGT-LF+ and DM-LF+ subjects. Statistical significance was determined by non-parametric Mann–Whitney *U* test and *p* < 0.05 was considered significant. *p < 0.05.

**Figure 6** shows the antigen-induced T-cell activation (expression of CD69) and Th polarization (Th1, Th2, Th9, and Th17) in the study groups. There was no significant difference in T-cell activation (as determined by CD69 expression) between the groups. However, in accordance to antigen-induced cytokine secretion, the percentage of Th9 cells was significantly higher in the DM-LF+ compared to the NGT-LF+ group. **Figure S4** shows the expression of Th polarizing transcriptional regulators (T-bet, GATA3, and ROR-γt) and immunomodulatory enzymes (Phox47 and HO-1) between diabetic and non-diabetic LF+ subjects. No significant difference was seen in the expression of T-bet, GATA-3, and ROR-γT between the groups. The expression of Phox47 and HO-1 was not significantly different between the groups.

## Discussion

In the present study, we elucidated the effect of diabetes on anti-filarial response, in LF+ subjects. Anti-filarial response is complex and multifaceted and involves both the innate and adaptive arms of the immune system (30). For innate immunity, we studied TLR signaling, and for adaptive immunity, we studied filarial antigen-induced responses. Under TLR signaling, we studied (1) secretion of cytokines (IFN-β, TNF-α, IL-6, IL-1β, GM-CSF, IL-10, TGF-β, IL-1Ra, IL-35, IL-27, IL-1Ra, IL-12, IFN-γ, IL-2, IL-33, IL-4, IL-9, IL-23, and IL-17) and chemokines (RANTES, MCP-1, IL-8, IP-10, and SDF-1); (2) upregulation of MHC (HLA-DR) and co-stimulatory molecules (B7-1/CD80 and B7-2/CD86); and (3) effector functions, which include secretion of defensins (α-Defensin-1) and upregulation of immunomodulatory enzymes (Cox-1, HO-1, IDO-1, and p47Phox). Under antigen-induced responses, we studied (1) secretion of cytokines (IFN-β, TNF-α, IL-6, IL-1β, GM-CSF, IL-10, TGF-β, IL-1Ra, IL-35, IL-27, IL-1Ra, IL-12, IFN-γ, IL-2, IL-33, IL-4, IL-9, IL-23, and IL-17) and chemokines (RANTES, MCP-1, IL-8, IP-10, and SDF-1); (2) Th immunophenotype (Th1, Th2, Th9, and Th17); (3) T-cell activation (upregulation of CD69); and (4) expression of master controllers of Th polarization (T-bet, GATA3, and RORγt). Our major findings were as follows: (1) Even in the absence of TLR- or antigen-induced activation, the basal level secretion of few cytokines was significantly altered in the DM-LF+ compared to the NGT-LF+ group. (2) TLR2 stimulation induced the secretion of TNF-α, IL-6, IL-1β, IL-10, IL-12, and IL-8; TLR4 stimulation induced the secretion of TNF-α, IL-6, IL-1β, IL-10, IL-27, IL-12, IL-23, IL-8, and IP-10; both TLR2 and 4 induced the secretion of α-defensin-1 and MCP-1 only in the DM-LF+ group. A significant difference was seen in the TLR2-induced secretion of IL-10, IL-1Ra, IL-17, and SDF-1 and TLR4-induced secretion of IL-9, IL-33, MCP-1, and SDF-1, between the NGT-LF+ and DM-LF+ groups. (3) TLR-induced expression of Cox-2 and IDO was significantly augmented by diabetes. (4) BmA stimulation induced the secretion of TNF-α, IL-6, IL-1β, IL-10, IL-27, IL-8, and SDF-1. In the presence of diabetes, the basal secretion of IL-1β, IL-27, IL-33, and SDF-1 is augmented while that of IL-10, IL-12, and IL-8 was downregulated. The augmented secretion of IL-9 in the diabetic group (DM-LF+) was associated with increased Th9 polarization. While we expected downregulation of many of these responses, most of these effectors were upregulated under diabetes conditions.

The basal level secretion of IL-12 and IL-8 was decreased while that of IL-1β, IL-27, IL-17, IL-9, IL-33, and SDF-1 was increased in the DM-LF+ compared to the NGT-LF+ group. This could be due to epigenetic modifications induced due to chronic hyperglycemia, onto the immune cells (31). It is well known that chronic hyperglycemia, as seen in diabetes, can induce epigenetic modifications onto immune cells, which can permanently alter their function, which is referred to as metabolic memory (32). TLRs serve as a first line of defense mechanism against pathogen invasion, linking innate and adaptive arms of the immune response (33). TLR2 polymorphisms were shown to be associated with bancroftian filariasis (34). In the present study, we studied TLR-induced secretion of (1) type 1 interferon (IFN-β), (2) pro-inflammatory cytokines (TNF-α, IL-6, IL-1β, and GM-CSF), (3) anti-inflammatory cytokines (IL-10, TGF-β, IL-1Ra, IL-35, IL-27, and IL-1Ra), (4) adaptive immune cytokines (Th1 cytokines—IL-12, IFN-γ, and IL-2; Th2 cytokines—IL-33 and IL-4; Th9 cytokines—IL-9; Th17 cytokines—IL-23 and IL-17), and (5) chemokines (RANTES, MCP-1, IL-8, IP-10, and SDF-1). Out of all the cytokines, chemokines, and defensins, diabetes specifically altered (1) TLR2-induced secretion of IL-10, IL-1Ra, IL-17, and SDF-1; and (2) TLR4-induced secretion of IL-12, IL-9, IL-33, MCP-1, and SDF-1. The expression of MHC (HLA-DR) and co-stimulatory (B7-1 and B7-2) molecules on monocytes, B cells, and granulocytes was largely unaffected. Finally, TLR2- and TLR4-induced expression of immunomodulatory effectors Cox-2 and IDO was significantly augmented by diabetes. Previously, it was shown that baseline expression of TLRs was significantly lower in B cells (but not in monocytes) of the LF+ subjects, compared to uninfected individuals (35). TLR stimulation of B cells from these subjects showed diminished activation and antibody secretion, indicating a state of immune unresponsiveness (35). Filarial parasite was shown to actively inhibit the expression and function of TLRs in human dendritic cells (36). In contrast, diabetes is largely a chronic inflammatory state that augments TLR expression and increases the activation in various cell types (37). Recently, fetuin-A was found to act as an endogenous ligand for TLR4 to promote free fatty acid-induced insulin resistance, in the adipose tissue (38). Several filarial antigens are known to directly bind to TLR4 and down-modulate their effect (39, 40). However, few filarial antigens were also found to augment TLR-induced inflammation, especially in macrophages and dendritic cells, indicating that not all filarial antigens have an anti-inflammatory effect (14, 15). Thus, under conditions of diabetes–filariasis comorbidity, one can expect either augmentation or inhibition of TLR responses, depending on the expression pattern of the filarial antigens, under the given set of conditions. In the present study, at least some of the TLR-induced responses were found to be augmented under conditions of diabetes–filariasis comorbidity (22). Interestingly, the TLR downregulation gets reversed when filarial patients develop chronic pathology, with augmented secretion of pro-inflammatory cytokines (41). They also show enhanced TLR-induced secretion of the pro-angiogenic factor, which promotes neo lymphangiogenesis leading to lymphedema and elephantiasis, a hallmark feature of lymphatic filariasis (42). As per the above model, diabetes can at least partially augment chronic pathology in LF+ subjects by enhancing TLR-mediated immune responses.

Compared to TLR-induced cytokine/chemokine secretion, the expression of immunomodulatory effectors like Cox-2, HO-1, and IDO-1 is poorly studied in filariasis and diabetes. These effectors produce second messengers like prostaglandin H, CO, and kynurenine, respectively, which have an immunomodulatory effect. In the present study, diabetes specifically augmented TLR-induced expression of Cox-2 and IDO in LF+ subjects. While Cox-2 expression induces a pro-inflammatory milieu, IDO expression induces an anti-inflammatory environment, as a counter mechanism (43). At least in a tumor microenvironment, this positive feedback loop augments inflammation and inhibits T-cell response, augmenting tumor growth and immune evasion, respectively (44). Whether the same strategy has been hijacked by filariasis and is augmented by diabetes, aiding pathology, is an interesting possibility that needs further exploration.

Next to TLRs, T cells play a vital role in orchestrating the immune response against filariasis. Antigen stimulation induced the secretion of pro-inflammatory cytokines (TNF-α, IL-6, and IL-1β), anti-inflammatory cytokines (IL-10 and IL-27), and chemokines (IL-8 and SDF-1). Out of all the cytokines, chemokines, and defensins secreted, diabetes specifically altered antigen-induced secretion of IL-12, IL-9, and SDF-1. T-cell-mediated immune responses during filariasis depend on the phase of the infection: (1) the acute phase is characterized by skewed Th2 response (with enhanced secretion of IL-4, IL-5, and IL-13); (2) the chronic phase is associated with “modified Th2 response” with Tregs (with enhanced IL-10 and TGF-β secretion) playing a major role compared to Th2 cells; and (3) the chronic pathology phase is characterized by a shift towards “Th1/Th17” (enhanced secretion of IFN-γ, IL-2, and IL-17) response, which takes place in individuals who develop pathology (45). Stimulation of T cells from LF+ subjects with *B. malayi* antigen showed impaired Th1 response with decreased expression of T-bet, Suppressor of cytokine signaling-1 (SOCS-1), SOCS-5, and SOCS-7 (27). Furthermore, *in vitro* culture of PBMCs from LF+ subjects with live larval stage 3 (L3) and microfilarial (mf) worms showed impaired expression of T-bet and GATA-3 and augmented expression of FoxP3 (46). In accordance with these reports, no significant Th1 or Th17 polarization was seen in LF+ subjects. While TLR-induced IL-12 and basal IL-33 secretion was augmented under diabetes condition, this was not seen at the level of Th1 and Th2 polarization. This indicates that, at least under *in vivo* condition, apart from IL-12 and IL-33, other factors might play a significant role in Th1/Th2 polarization.

Compared to TLR stimulation, antigen stimulation induced the secretion of only pro-inflammatory cytokines and chemokines. Compared to Th1, Th2, and Th17 cell types, Th9 cells were recently characterized and were found to be involved in allergies, autoimmunity, and tumor evasion (47, 48). Their role in both diabetes and filariasis is largely unknown. Recently, expansion of filarial antigen-specific Th9 cells in whole blood culture was found to be associated with chronic pathology (49). In the present study, diabetes augmented IL-9 secretion and increased Th9 population. Like IL-9, the basal level of SDF-1α was found to be significantly high in LF+ subjects with diabetes. Previously, SDF-1/CXCR4 was found to promote chronic pathology in LF+ subjects (50). Thus, diabetes, by augmenting IL-9 and SDF-1, can promote chronic pathology in LF+ subjects.

In conclusion, in the present report, the effect of diabetes on TLR pathway and antigen-induced immune response in LF+ subjects was studied. Several parameters including cytokines and chemokines, antigen processing and presentation molecules, T-cell activation marker, Th polarization, expression of master regulators of Th polarization, and immunomodulatory effectors were studied. The most interesting and unexpected finding of this study is that, while we expected downregulation of TLR and antigenic responses, diabetes had a modest but significant augmenting effect on specific TLR-mediated inflammatory markers. Most of the inflammatory markers that were found to be augmented by diabetes have previously been shown to promote chronic pathology in LF+ subjects (51, 52). The major limitations of this study are the limited sample size and the cross-sectional study design, which makes it impossible to decipher the cause–effect phenomenon. However, the major strength of the study is that it was conducted in a filarial endemic population wherein the prevalence of diabetes is rapidly increasing (53). Overall, diabetes was found to augment certain inflammatory components that can promote chronic pathology in LF+ subjects, and this needs further validation in a large cohort study.

## Data Availability Statement

The raw data supporting the conclusions of this article will be made available by the authors, without undue reservation.

## Ethics Statement

The studies involving human participants were reviewed and approved by the MDRF Institutional Ethical Committee. The patients/participants provided their written informed consent to participate in this study.

## Author Contributions

VM, SB, and VA conceived and designed the experiment. JS and SM performed the experiment. JS and VA analyzed the data. JS and VA drafted the manuscript. All the authors contributed to the discussion and reviewed the manuscript. VA is the guarantor of the data. All authors contributed to the article and approved the submitted version.

## Funding

The Department of Genetics, University of Madras, has received funds for infrastructural support from DST-FIST and UGC-SAP programs. The funders had no role in study design, data collection and analysis, decision to publish, or preparation of the manuscript.

## Conflict of Interest

The authors declare that the research was conducted in the absence of any commercial or financial relationships that could be construed as a potential conflict of interest.

## Publisher’s Note

All claims expressed in this article are solely those of the authors and do not necessarily represent those of their affiliated organizations, or those of the publisher, the editors and the reviewers. Any product that may be evaluated in this article, or claim that may be made by its manufacturer, is not guaranteed or endorsed by the publisher.

## Supplementary Material

Supplementary Figure 1 Gating strategy used for the analysis ofHLA-DR, B7-l(eD80), B7-2 (eD86) on Monocytes and Granulocytes and B cells. FSe vs SSe plot showing monocyte, lymphocyte and granulocyte gates **(A)**. FSe-A vs FSe-H plot showing gated single cells oflymphocytes **(B)**, monocytes **(C)**, Granulocytes **(D)**. CD19 vs FSC-H plot showing the gated CD19+ B cells **(E)**. CD14 vs FSC-H plot showing the gated CD14+ Monocytes **(F)**. Expression of HLA DR on lymphocytes **(G)**, monocytes **(H)**, Granulocytes **(I)**. Expression of B7-1 (CD80) on lymphocytes **(J)**, monocytes (K), Granulocytes **(L)**. Expression ofB7-2 (CD86) on lymphocytes **(M)**, monocytes **(N)**, Granulocytes **(O)**.

Supplementary Figure 2 Gating strategy used for analysis of T helper cell subtype analysis. FSC *vs* SSC plot showing the gated lymphocyte population **(A)**. FSC-A *vs* FSC-H plot showing gated single cells of lymphocytes **(B)**. CD3 *vs* CD4 plot showing the gated CD3+CD4+ T helper cells **(C)**. IFN-y *Vs* IL-4 plot showing the gated Thl and Th2 population **(E)**. IL-9 *Vs* IL-17 plot showing the gated Th 17 and Th9 population **(F)**.

Supplementary Figure 3 Effect of diabetes on the TLR induced expression of MHC and costimulatory molecules in LF+ subjects.

Supplementary Figure 4 Effect of diabetes on expression of Th master regulators and immunomodulatory enzymes in LF+ subjects.
