# Supplementary figures and images for: Augmented Innate and Adaptive Immune Responses Under Conditions of Diabetes–Filariasis Comorbidity
Source: Front Immunol. 2021 Sep 10;12:716515. doi: 10.3389/fimmu.2021.716515 (PMC8462934; doi:10.3389/fimmu.2021.716515)

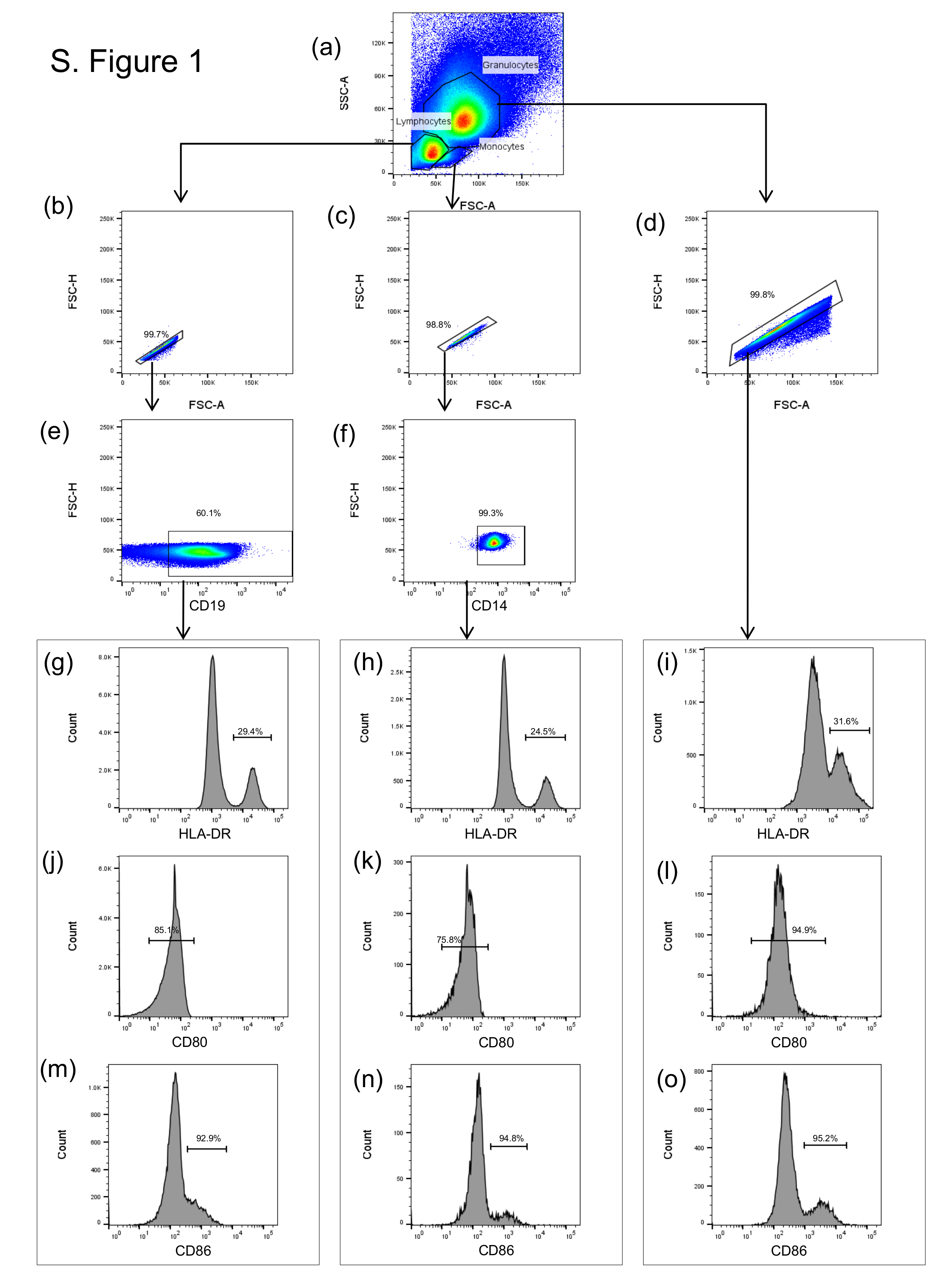

Supplement: Supplementary Figure 1 — Gating strategy used for the analysis ofHLA-DR, B7-l(eD80), B7-2 (eD86) on Monocytes and Granulocytes and B cells. FSe vs SSe plot showing monocyte, lymphocyte and granulocyte gates (A). FSe-A vs FSe-H plot showing gated single cells oflymphocytes (B), monocytes (C), Granulocytes (D). CD19 vs FSC-H plot showing the gated CD19+ B cells (E). CD14 vs FSC-H plot showing the gated CD14+ Monocytes (F). Expression of HLA DR on lymphocytes (G), monocytes (H), Granulocytes (I). Expression of B7-1 (CD80) on lymphocytes (J), monocytes (K), Granulocytes (L). Expression ofB7-2 (CD86) on lymphocytes (M), monocytes (N), Granulocytes (O). [file Image_1.tif]

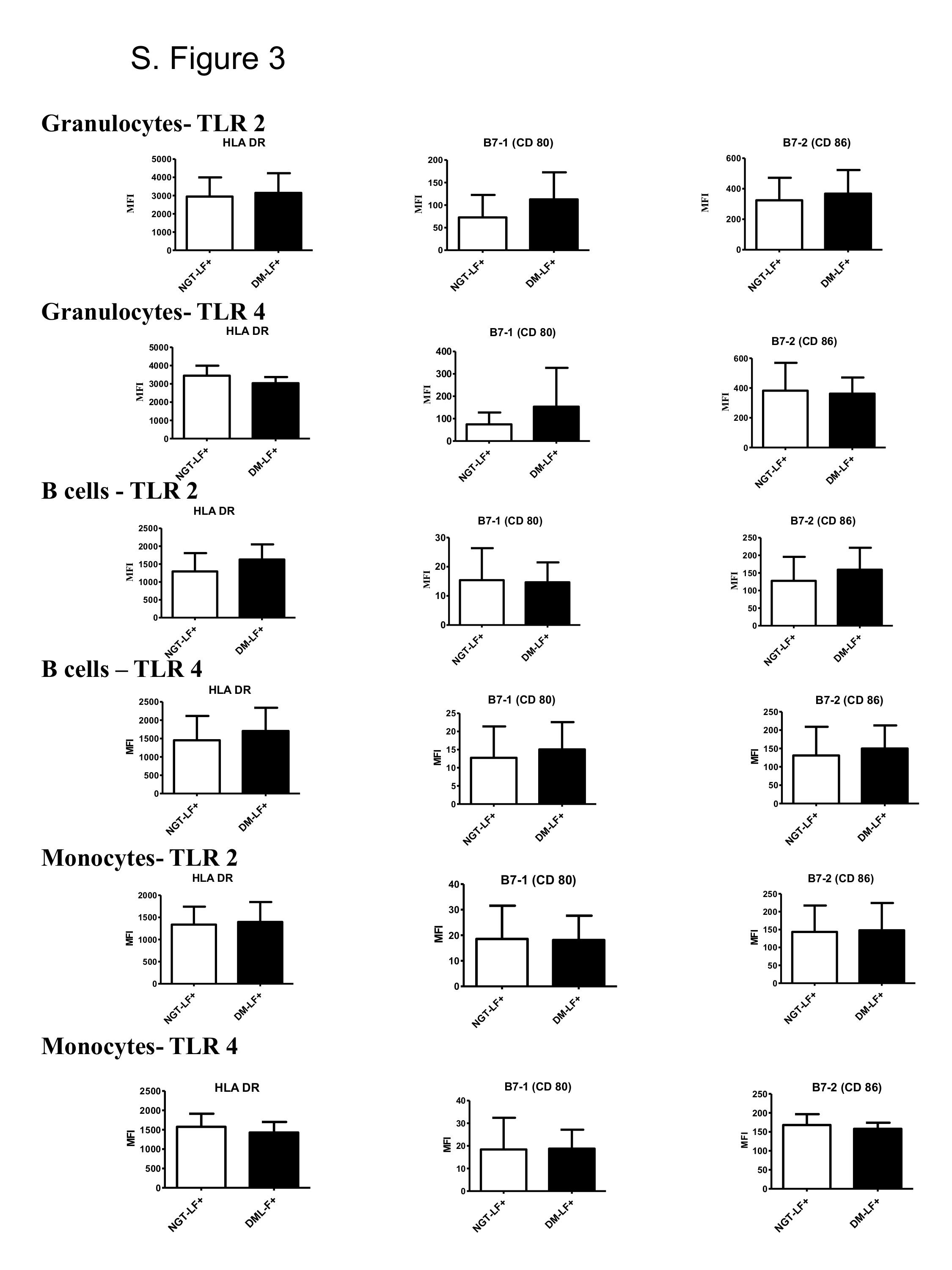

Supplement: Supplementary Figure 2 — Gating strategy used for analysis of T helper cell subtype analysis. FSC vs SSC plot showing the gated lymphocyte population (A). FSC-A vs FSC-H plot showing gated single cells of lymphocytes (B). CD3 vs CD4 plot showing the gated CD3+CD4+ T helper cells (C). IFN-y Vs IL-4 plot showing the gated Thl and Th2 population (E). IL-9 Vs IL-17 plot showing the gated Th 17 and Th9 population (F). [file Image_2.tif]

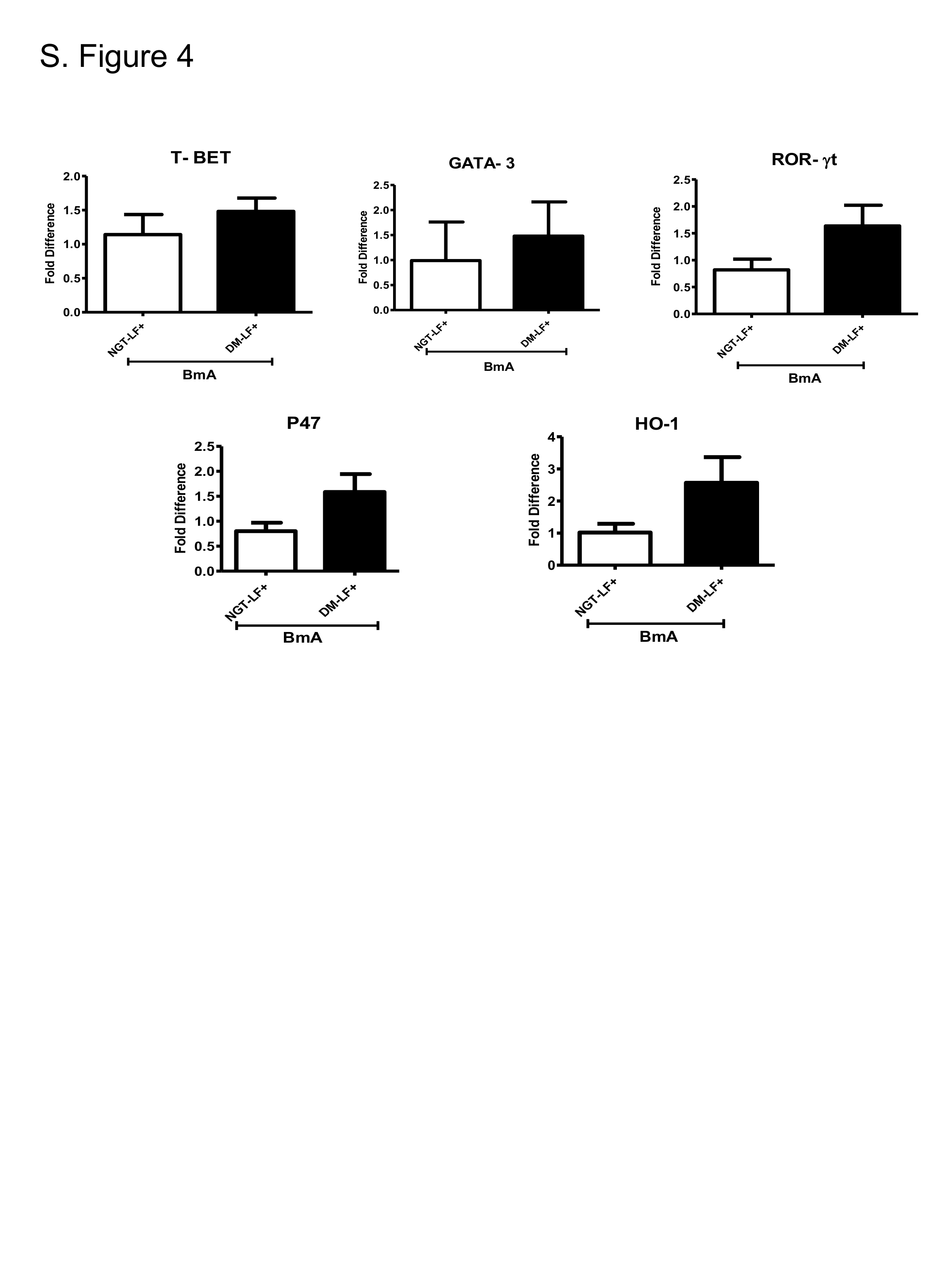

Supplement: Supplementary Figure 3 — Effect of diabetes on the TLR induced expression of MHC and costimulatory molecules in LF+ subjects. [file Image_3.tif]

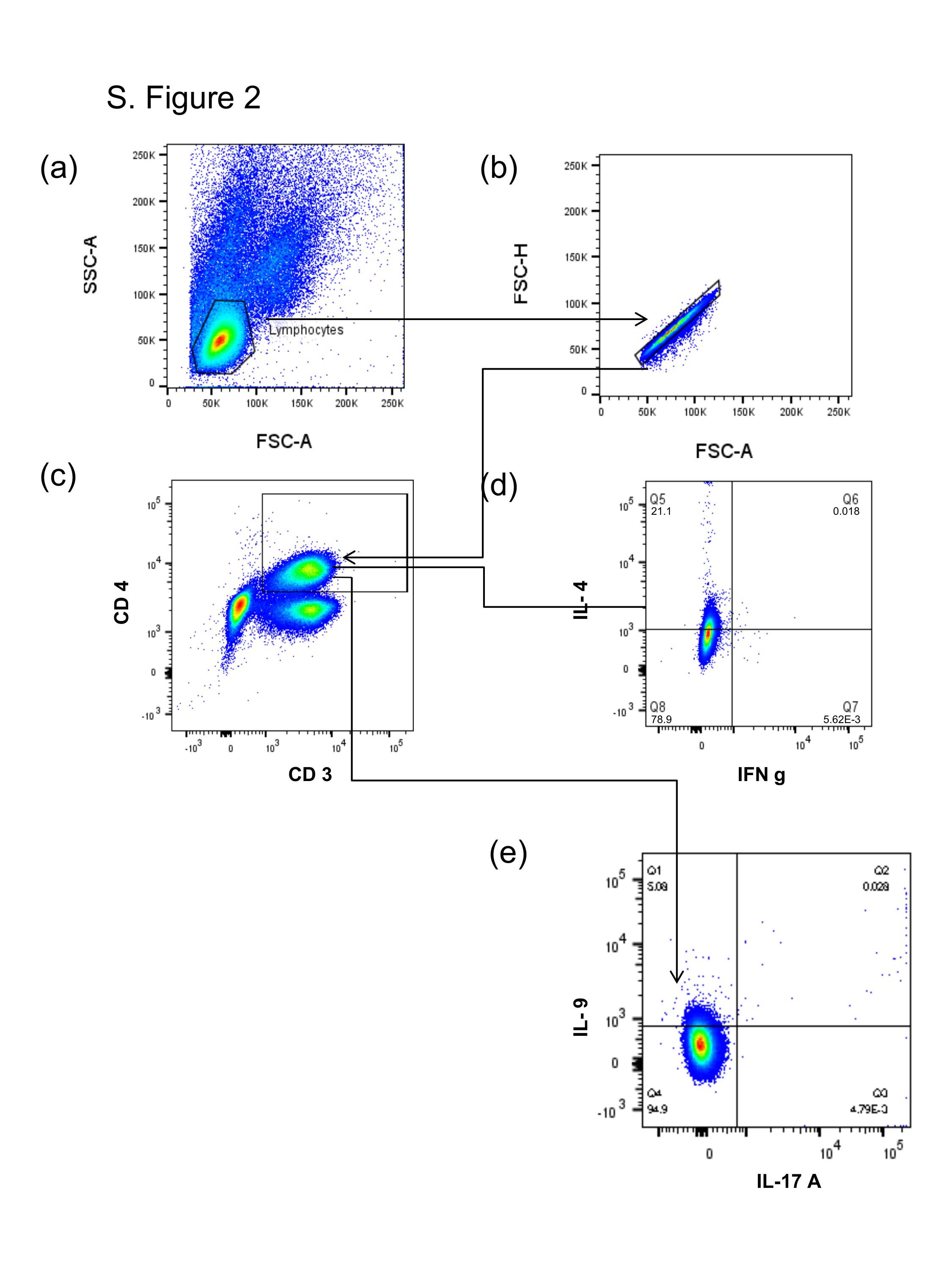

Supplement: Supplementary Figure 4 — Effect of diabetes on expression of Th master regulators and immunomodulatory enzymes in LF+ subjects. [file Image_4.tif]
